# Biomechanical Principles and Techniques—A Systematization for Sport Climbing

**DOI:** 10.3390/jfmk11010103

**Published:** 2026-02-28

**Authors:** Silas Dech, René Kittel

**Affiliations:** 1 Division “Climbing Active”, Brandenburg Association for Health Promotion e.V., 14467 Potsdam, Germany; 2 German Federation of Climbing Therapy “FAKT! e.V.”, 14473 Potsdam, Germany; 3 Training and Movement Science, Department of Sports and Health Sciences, University of Potsdam, 14469 Potsdam, Germany

**Keywords:** sports biomechanics, sports technique, sports performance, human performance optimization, climbing, bouldering

## Abstract

**Background**: Sport climbing, encompassing lead, bouldering, and speed disciplines, has transformed from a niche activity to a widely popular trend, notably after its Olympic debut at the Tokyo Games 2021. This recognition spurred an increase in publications. Despite the emerging scientific interest, terminology in climbing textbooks often relies on experiential rather than scientific understanding, leading to inconsistencies. This paper aims to standardize terminology by applying sports science frameworks, including biomechanics, training science, and sports medicine. **Methods**: The study reinterprets general sports science concepts for climbing-specific applications, proposing a structure of climbing skill that covers physical fitness components, biomechanical principles and techniques (body positioning), and specific components (hand and foot positioning). This integrated approach seeks to establish a coherent nomenclature, facilitating research, training, prevention, and rehabilitation within the climbing discipline. **Results**: Five primary climbing principles are proposed: optimal wall contact, maintained stability, center of mass shift, movement initiation from the legs and optimal climbing speed. Two technique categories—frontal and rotational—are defined in consideration of the spatial position of the pelvic frontal plane in relation to the wall surface. Each climbing technique can be described by applying the three-phase model of acyclic movements. Principles and techniques both aim to maximize efficiency in moving and resting on the climbing wall. **Conclusions**: A unified understanding of climbing principles and techniques is vital for progressing research, training programs, prevention strategies, and rehabilitation efforts in sport climbing. Adopting a comprehensive sports science framework promises enhanced clarity and efficacy in climbing practices, benefiting both theoretical analyses and practical applications.

## 1. Introduction

Sport climbing has been practiced for over 150 years, beginning with the first ascent of the Schandau Tower in Saxon Switzerland in 1864. The first climbing competition occurred in 1985 on natural rock in Bardonecchia, Italy. In 1989, the “Rockmaster” in Arco introduced lead climbing as the first World Cup event on an artificial wall with manufactured holds. Thirty years later, the climbing disciplines of speed, lead and bouldering were combined as the Olympic sport of climbing at the Tokyo Games, leading to a significant boom in these disciplines [1]. Prior to this boom, climbing-specific terms and content were established based on the experiences of top climbers like Wolfgang Güllich and John Shermann [2,3]. This boom has greatly increased the number of climbing-related books aimed at diverse audiences such as boulderers, climbers, adults, youth and children [4,5,6]. Some publications focus on teaching [7,8,9], while others use wall structure and inclination as organizational bases [2,10]. Additional books incorporate sports science disciplines like kinesiology, training science or sports medicine [11,12,13], contributing to inconsistencies in climbing terminology.

Climbing’s scientific relevance has grown, focusing on finger flexor strength as a performance-limiting factor [14,15]. Consequently, maximal strength and endurance tests are predominantly used in performance diagnostics [16,17]. However, climbing also requires efficient body positioning and movement on the wall to reveal minimum expenditure of energy and to reduce stress on the performance-limiting finger flexor muscles. Based on biomechanical principles, specific techniques are used in this regard. Climbing technique is, therefore, a practical determinant of performance [18,19,20,21]. In many systematic reviews, climbing technique is overlooked as a latent variable [22,23,24], possibly due to difficulties in testing and measurement [25]. In a study of 30 athletes, it was concluded that climbing technique accounts for 33% of climbing performance [26]. Detailed methodological descriptions were not given. Another study with three participants used video-based expert ratings to assess specific climbing techniques, such as drop knee, heel hook, and rear flag [27]. These assessments used 5–6 sub-steps of the movement process based on Hörst’s descriptions (2008). Intra-expert agreement (reliability) ranged from 74% to 94%.

Despite limited evidence directly linking climbing technique and performance, associations exist between years of climbing experience [28] or self-assessed climbing level [29] and reduced oxygen consumption during submaximal climbing. These variables are likely connected to various performance components, including climbing technique [30]. Despite the surge in popularity and research, there is no unified framework for categorizing climbing techniques, which complicates research efforts and leads to fragmented findings. In this regard, Augste et al. state [31], “Climbing technique [as a performance determinant] is currently neither systematically classified from a practical nor from a scientific perspective” (p. 28, own translation).

Due to the lack of systematization, various aspects are assigned to the “technical field”, including balance, coordination, accurate footwork, slab climbing technique, overhanging technique, weight and body position transfer [19], static or dynamic climbing styles [32] and optimizing the trajectory path of the center of mass [33]. Physical qualities, techniques, principles, and other components are mixed up here. In German-speaking regions, “technical climbing” often refers to aid climbing, meaning climbing with aids such as rope ladders, hooks, and ropes, as opposed to free climbing. The absence of systematization complicates performance diagnostics, considering, e.g., the implementation of specific climbing principles or sport climbing techniques. In the development process for a scientifically based performance diagnostic in sport climbing, the technique test was discontinued due to a lack of economy and validity [31]. Consequently, the technique needed to be excluded from the performance diagnostics manual [34]. The need for a robust evaluation remains critical. Besides performance diagnostics, the economical climbing technique is linked to injury prevention [35,36] and can be utilized in climbing therapy [37]. A coherent sport climbing terminology supports the practitioners, scientists and therapists targeted through this paper’s systematic framework proposal.

## 2. Methods

To propose a sport climbing terminology, sports science concepts are reevaluated and tailored specifically for the intricate demands of climbing. The structured framework is organized from general to specific aspects of the climbing skill based on a sports science perspective. It encompasses physical fitness components, biomechanical principles and techniques (body positioning), and their specific components (hand and foot positioning). Anthropometric components are neglected here.

### 2.1. Systematization of Climbing Skill

Climbing, as one type of fundamental learned human movement and skill, relies on all physical fitness components (including strength) as general determinants for athletic success [38,39,40]. Skill-related components like balance, coordination or technique complement performance capacity on a sport-specific level. Furthermore, general biomechanical principles are applicable for enhanced performance, e.g., the principle of temporal coordination of partial impulses [41]. These principles represent universal laws governing movement. They are complemented by sport-specific ones. In respect of climbing, such fundamental rules concern body positioning and movements when engaging with gravity; they form the foundation of climbing techniques.

**Figure 1:**
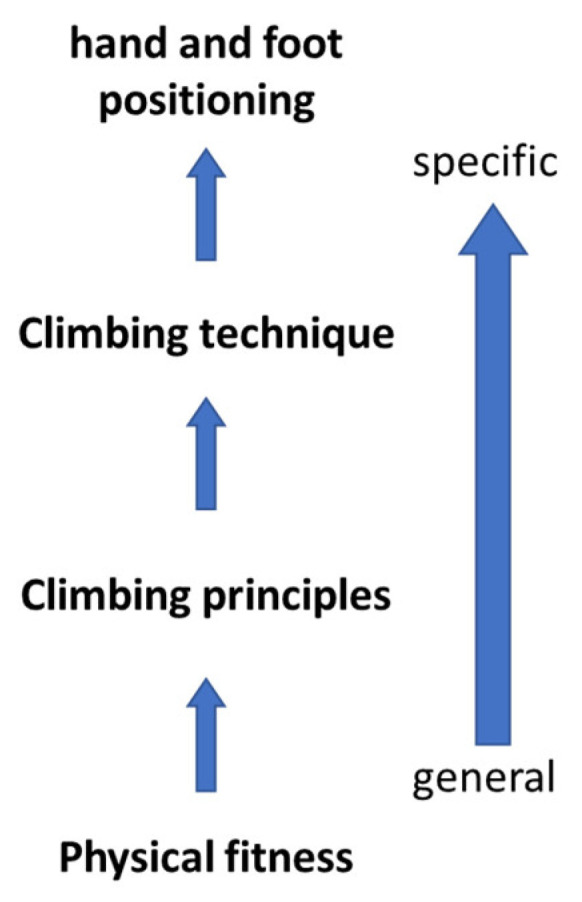
Systematization of climbing as a skill from general to specific.

In contrast, climbing techniques describe specific movement sequences [42] and follow the three-phase model of acyclic movements (see Section 2.2) [43]. Techniques offer solutions for specific “movement-problems” to enable progress on the wall. This, in turn, involves more specific components such as footwork or gripping types, which by themselves do not constitute a climbing movement. However, they do facilitate contact with the climbing wall as a prerequisite for force transmission and counteracting gravity. Figure 1 summarizes this system from general to specific aspects.

Before the main climbing principles, techniques, and specific components are elaborated, the structure of climbing movements is clarified as a basis.

### 2.2. Climbing Movements—Three-Phase Model

**Figure 2:**
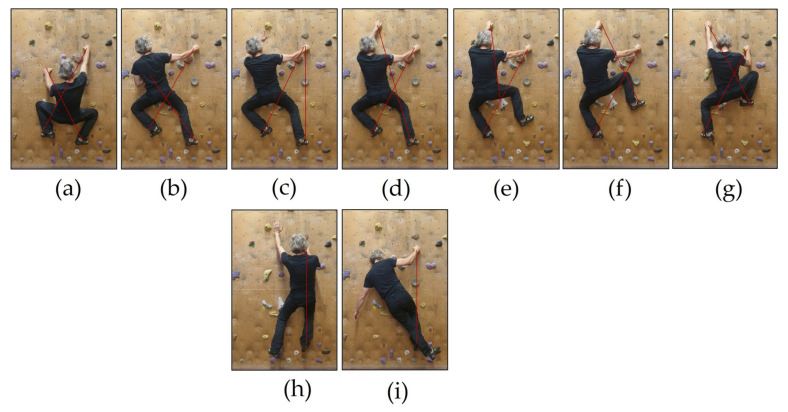
The photographs show a sequence of natural frontal gripping (**a**–**d**) and stepping (**e**,**f**) techniques with (**a**) starting position (X-position); (**b**) phase 1: preparation CoM-shift (intermediate X-position); (**c**) phase 2: gripping (Λ-position); (**d**) phase 3 merged into phase 1 for the following stepping technique: intermediate stabilization (shifted X-position); (**e**) phase 2: stepping (Y-position); (**f**) intermediate stabilization (shifted X-position); (**g**) phase 3 stabilization (X-position); and (**h**,**i**) I-positions during phase 2 with (**h**) during fast ladder climbing; and (**i**) during back flagging.

When routes or boulders are climbed, it becomes evident that the single movements are distinctly different and thus, from a sports science perspective, are acyclic. Consequently, a climbing movement is divided into three phases according to the general model of acyclic movements [43]: preparation phase = CoM (Center of Mass) shifting; main phase = hand and/or foot placement; and final phase = stabilization [4,8,13]. This model applies to all climbing movements, with underlying climbing principles. Within this structure, single climbing positions can be visualized using letters. This gives rise to a climbing alphabet in kinesiology [7,44]. A stable starting position serves as the foundation for phase 1 and is typically depicted by the Χ-position. The Greek capital letter Chi (Χ) visualizes the diagonal connections between the usually four contact points on the wall (Figure 2a). To prepare for the climbing movement, the CoM is shifted so that either a hand or a foot can be released from a hold. In phase 2, at least one extremity is placed on a new hold. During this process, not more than three extremities remain in contact with the climbing wall [5,7]. Thus, wall contact is reduced from four to three points (body parts apart from hands and feet are neglected for simplification). Based on the connection lines of loaded holds, this results in the Greek capital letters Lambda (Λ) when placing a hand (Figure 2c) and Upsilon (Υ) when placing a foot further (Figure 2e).

In some movements, the three-point rule is bypassed. If only two contact points are maintained on the wall, such as in Figure 2h,i, the connection line forms the Greek letter Iota (Ι). In very special cases, there might be only one or even no contact point, such as in climbing movements like campusing or run’n’jump, respectively. A climbing movement is finalized according to the model by stabilizing (phase 3). This serves then as the starting position for the next movement.

Even modern climbing movements, like a double dyno, can be structured into (1) preparation, (2) execution, and (3) stabilization phases. When multiple climbing movements are executed consecutively (fluid climbing through momentum conservation), phases 3 and 1 might merge similarly to cyclic sports. The Three-Phase Model of climbing movements is used to relate biomechanical principles to specific climbing phases and to describe sport climbing techniques in the following.

### 2.3. Biomechanical Climbing Principles

Climbing principles are applicable to most climbing movements. From a sports science perspective, they are based on the physical laws of dynamics with fundamental quantities such as length (l in meters), time (t in seconds) and mass (m in kilograms), and their derived quantities like velocity (v in m/s), force (F in N) and acceleration (a in m/s^2^), and their effect on the body [41]. There are cross-disciplinary [45] and sport-specific principles [42]. Cross-disciplinary principles can only be partially applied to the specific movement demands of a specific sport. Particularly in climbing, with its countless degrees of freedom, a separate consideration seems necessary. The application of climbing-specific principles aims to increase the efficiency of climbing movements. However, the terminology in the literature is inconsistent. In some publications, those aspects are traditionally categorized as principles from a sports science perspective [42]. Others classify them as a technique, technical foundations [3,4,6,13] or movement characteristics [8] without a structural relation [2].

A review of the climbing-specific literature also reveals that a multitude of terminologies are used for similar functions. Sometimes, similar movement characteristics, such as the principle of climbing with a straight arm and the principle of climbing with a lowered CoM and close to the wall, are termed differently. Furthermore, climbing principles such as the principle of “deadpointing” are hierarchically equated to the principle of “dynamic climbing” [3]. The aim is to develop a structure with main principles and assign corresponding sub-principles, thereby proposing a sports science structure.

## 3. Results

### 3.1. Main Climbing Principles

Five main climbing principles are proposed: Optimal Wall Contact; Maintained Stability; Center of Mass Shift; Movement Initiation of the Legs; and Optimal Climbing Speed. Each one is addressed point by point in detail and exemplary sub-principles are given.

#### 3.1.1. Principle of Optimal Wall Contact

This principle derives from mechanical engineering, where various components are interlocked to transmit forces. In a form-fit connection, the parts should fit together precisely. In a force-fit connection, for example, pressure is applied to build friction or create a clamping connection. These connection principles are equally applied in climbing. Therefore, body parts (e.g., hands and feet) should establish optimal contact with the wall (hold). The contact can be form-fitted [3,8,46] and force-fitted through friction or clamping. Thus, the principle of optimal form-fit is subordinate to the principle of optimal wall contact. A selection of specific contact possibilities is presented in Section 3.3 (hand and foot positioning).

Optimal wall contact is characterized not only by form-fit but also by the magnitude of force applied to the grip, which should only be as high as necessary. In climbing, this is referred to as “soft gripping” [8] and is therefore a sub-principle. A study by Fuss and Niegel (2008) showed that experienced climbers demonstrated smaller contact forces, shorter contact times, smaller impulses, higher friction coefficients and more consistent force-time patterns compared to less experienced climbers [47].

Foot placement should also be loaded optimally. Using “silent feet” [8] is often referenced. This sub-principle aims for efficient footwork and is crucial for optimal wall contact. As with grips, the shape of the foothold combined with the orientation of the contact surface dictates which type of step/foot position enables optimal form-fit or force-fit. If a foothold is angling away from the climber, optimal force transfer typically occurs through pulling on the foothold by using a heel or toe hook. Thus, this principle is a prerequisite for hand and foot positioning.

#### 3.1.2. Principle of Maintained Stability

The physical foundation of this principle is the law of inertia (Newton’s first law). It states that a body is in equilibrium when the sum of all acting forces is zero. Achieving force equilibrium is the goal when applying this climbing principle. It dictates that the partial body masses during climbing or holding positions should be arranged such that the overall body mass (Center of Mass, CoM) remains stable and does not tilt away from the wall (climbing problem of the “barn door”). If not maintained, additional force would be needed to fix the position, which would be inefficient. Wick (2013) describes stable and unstable equilibrium states in this context [45]. In climbing-specific terms, phases 1 and 2 of the climbing movement require dynamic (unstable) equilibrium, and phase 3 requires static (stable) equilibrium. From an efficiency standpoint, the gravitational force components must be balanced to maintain stability during climbing.

Stability on the climbing wall can be achieved through various options, explaining the use of different terms to describe the same biomechanical phenomenon. Through the contact points (e.g., hands, feet), a body position must be adopted where the CoM acts as the point of gravity perpendicular to the support area. The various applications of this principle are structured based on the extremities in contact with the climbing wall.

Before preparing a climbing movement (Phase 1), the starting position is stable when the CoM is vertically aligned between both holding hands and falls onto the support area between both feet (Χ-position, Figure 2a). In the second phase of a climbing movement, placing a hand (Λ-position, Figure 2c) or placing a foot (Υ-position, Figure 2e), stability is usually maintained through three contact points on the wall. This form of stability is referred to as the principle of three contact points [42]. Crucial for efficiency is the CoM’s position relative to the three contact points. Typically, for efficient and stable distribution of partial body masses across the three contact points on the wall, one hand and its contralateral leg are loaded mainly (diagonality) [42]. Diagonality is standard in many movements. For example, while running, the left arm swings forward in accordance with the right leg. Vertical climbing is comparable to crawling. Biomechanically, the force vector pulls from the main loaded hand and pushes from the opposite foot (mainly loaded foot) towards the body center. The other (supportive) foot and hand are loaded less.

Even when climbing positions are adopted with only two contact points, the principle of maintained stability is essential for efficiency, too [48]. Depending on the specific climbing task, diagonality is emphasized (e.g., standard flag) or negated (e.g., fast ladder climbing, outside/inside flag) [8]. Despite lacking diagonality, the effort remains efficient if, during movement, the CoM is vertically aligned over the main loaded leg, e.g., fast ladder climbing, when a hand and ipsilateral foot move simultaneously (I-position, Figure 2h). While back-flagging (Figure 2i), I-position is achieved by crossing the free leg behind the loaded one and balancing in this way, so that CoM is above the foothold. This variation in the I-position is more efficient in overhangs.

Ignoring the principle of maintained stability might result in the climbing problem “barn door”; for example, in a climbing situation where both hands reach far beyond the side of the loaded foothold or during the two-point stability form if the CoM lies outside the connecting line of the contact points. Particularly in overhang climbing, the body position on the climbing wall is likely to become unstable, swings uncontrollably away from the wall like a barn door opening. Instability always occurs if the gravitational force, especially its component perpendicular to the wall, is not balanced.

#### 3.1.3. Principle of Center of Mass Shift

This principle is based on cross-disciplinary principles of counteraction, initial force, and optimal acceleration path. Additionally, it encompasses several distinct aspects throughout the course of a climbing movement. The direction and moment (climbing phase) of the CoM-shift must be considered. The goal of the preparation phase of a climbing movement (Phase 1) is to release one extremity through a Υ-, Λ-, or Ι-position. This typically occurs through horizontal CoM-shifts towards the wall, stabilizing the force equilibrium for the upcoming release of one extremity. In traverse climbing, the preparatory movement can initially be opposite to the climbing direction. Dynamic moves are also initiated with an opposite preparatory motion, which is in line with the general biomechanical principle of initial force [41]. In a large upward movement (e.g., jump), initiation proceeds against the jump direction and, if possible, away from the wall [42]; further adhering to the general principle of the optimal acceleration path.

CoM-shift in the direction of motion is inherently the goal of phase 2 of a climbing movement. In phase 3 of a climbing movement (stabilization) [4,13], the CoM is lowered and brought close to the wall. Ideally, the arms are extended. Essential finger flexor muscles establish grip contact with the climbing wall to prevent falling. Bent arms engage additional elbow, shoulder and shoulder girdle muscles [49], which are less necessary to transfer force to the grips in this specific phase. Furthermore, overhead grip with extended arms was recently found to lower handgrip strength asymmetries [50]. A lowered CoM is merely one feature for the fourth principle.

A CoM close to the wall combines three biomechanical aspects for an efficient climbing position and reduces finger flexor stress. Firstly, a reduced distance (lever arm) decreases the torque applied to stay on the wall. Secondly, gravitational force components can be increasingly counteracted through footwork (leg muscles). Thirdly, the direction of force applications at the holds is optimized, which increases the normal force and thereby the friction force, counteracting slippage. The biomechanical sub-principle of a low CoM-distance to the wall was found to be positively associated with ranks in a climbing competition [51].

#### 3.1.4. Principle of Movement Initiation from the Legs

This principle pertains to the muscular engines responsible for generating lift work. Control and displacement of the CoM in all three planes can occur through the work of the upper extremities, the lower extremities or mixed efforts involving the optimal coordination of partial impulses with the subordinate aspect of optimal kinetics (large moving muscles) and modulation (small fine-tuning muscles) for whole-body movements [45].

It is widely recognized that leg and hip musculature can generate more and longer-lasting physical work than the muscles of the upper extremity. This is supported by current records: a squat with 490 kg by Ray Williams; 30,000 reps in 24 h by Andrei Rosu versus a pull-up with 104.55 kg by David Marchante; 11,900 reps in 24 h by Lennert Schots. This insight is reflected in the current principle. Climbers either face maximal strength requirements like in bouldering or mixed requirements from maximal strength, strength endurance to endurance in route climbing. For both scenarios, it is economical if the initiation of movement primarily involves the leg musculature. Options include pushing actions like stepping, stemming and rocking over or hooking for movement initiation, not stabilization purposes. This gives rise to the classic climbing expression, preferring squats over pull-ups, or in modern terms: “Magic comes from the legs”.

In jumps (dynos), it has been demonstrated that successful jumps exhibit higher vertical take-off velocity than unsuccessful ones, with the force generated by the lower extremities being 1.8 times greater than that of the upper extremities [52]. This does not mean the upper extremities do little or no work. They correct movement control or provide the final impulse according to the general principle of temporal coordination of partial impulses. Exceptions are movements like campusing, where the legs are primarily used for initiating/facilitating momentum and stabilizing.

#### 3.1.5. Principle of Optimal Climbing Speed

This principle translates the general principle of optimal acceleration tendency to climbing situations, simultaneously establishing a connection to cybernetics (motor control). In climbing, there is a constant situational need to decide whether to move slowly and control for maximum precision or quickly for maximum time efficiency, which requires high coordination demands. The former is known as “static climbing” and the latter as “dynamic climbing”. Movement speed of individual extremities or body parts can also be associated with this principle: for example, a hand moves slowly or quickly snaps to the next hold. Some modern climbing techniques are inherently executable only quickly, e.g., dyno or flick. Therefore, the classification of dynamic vs. static climbing is often associated with technique [3,4,13,53]. Others classified it as being associated with movement quality or style [2,6]. Here, it is proposed as a superordinate principle since movement speed can be applied across most techniques. A frontal climbing movement can be executed slowly on very small (foot) holds or quickly on large ones with wide distances, depending on the requirement.

For classification (fast or slow) the regulatory model of either an open-loop or closed-loop control system can be used [54,55]. If movements are slower than 150–200 ms, a desired vs. actual value comparison between the movement goal and the actual result is possible. A planning error allows correction through feedback, categorizing these movements as static climbing. If movements are faster, no feedback-driven change is possible [56]. The motor program triggering the movement must be accurate; otherwise, the climbing movement will not be completed successfully. During faulty execution, correction is impossible during movement, resulting in failure to reach the movement goal.

The authors further distinguish dynamic movements into dynamic (1) and dynamic (2). Both movement forms are open-loop controlled but differ in movement scope and coordination demands. To structure the spectrum from a “simple” grab to a paddle-dyno or run’n’jump, the authors consider reversibility and the number of extremities involved in the movement. Dynamic (1) implies that movement failure prevents reaching the goal, but the start position can be stabilized for another attempt. Often, only one extremity is used, such as in quick snaps. Dynamic (2) offers no margin for error in execution; the climber falls and must restart from the ground. At least two extremities move simultaneously, e.g., run’n’jump, paddle-dyno.

When climbing movements are executed dynamically, clarification is needed on where to accelerate and when to grip. The direction of acceleration is explained by the principle of CoM displacement. The magnitude of acceleration and timing of hand placement are explained by the deadpointing model. This is a sub-principle of the optimal climbing speed principle, applicable only to dynamic movements.

The climbing literature similarly describes the body or parts of it being accelerated along the climbing wall, with grip occurring at the moment of motion reversal. This moment is called the dead point, marking where gravity counteracts movement energy, momentarily stabilizing the body (Newton’s First Law F = 0). Contrary to physics, Fuss and Niegl’s experimental study found that success is more likely when the target is overshot and the hand is placed just ahead of the dead point (pre-deadpointing) [52]. According to this study, the sub-principle “deadpointing” should be referred to and implemented as the “overshooting and pre-deadpointing.”

### 3.2. Climbing Techniques

A sporting technique is a pattern of movement appropriate for a skill like climbing [40]. Such techniques have to be learned in order to increase efficiency [57]. In relation to sport climbing, this means that a climbing-specific problem (handhold-foothold pattern) is solved with a targeted movement. In a complete climbing movement, all extremities are placed at a new contact point of the wall, whereby CoM is shifted, e.g., upwards. For that, one (e.g., jump) or several climbing techniques (e.g., a sequence of natural frontal gripping and stepping) are possible. Similarly to sports games, there are often several practical solutions. Even though different techniques can be effective, not everyone is efficient. For specific handhold and foothold arrangements in particular wall inclinations, specific efficient movement patterns have been established.

Climbing techniques include all partial body changes (technique characteristics) aiming to release and/or move at least one extremity in between. They are based on climbing principles and always end with an (intermediate) stabilization phase according to the three-phase model (see above). Figure 2 shows a sequence of two natural frontal techniques for gripping (a–d) and stepping (e–g). For those techniques, the sub-principle of a three-point wall contact is used. To complete a full climbing movement, the left foot and right hand need to be displaced, too. In the photographs, you can see that these extremities always stay in the same holds. In Figure 2h,i, a two-point of contact solution of the same handhold–foothold pattern is given. Furthermore, in competitions, single climbing “moves” can be relevant. Reaching the zone or top hold in bouldering serves for higher scores. In lead, even the attempt to reach a higher hold increases the score. In speed discipline, the consideration of individual moves only plays a role in performance analysis or training.

#### 3.2.1. Categorization of Climbing Techniques

In the dissertation of Low (2005) [58], climbing techniques were classified according to the position of the ipsilateral foot in relation to the reaching hand: toe, outside and inside edge. If the foot is in the air, it is referred to as the “air technique,” which is equivalent to the I-position. Sensors in shoe soles can provide biomechanical measurement parameters. However, since feet have various points of contact with the wall while climbing (see below), it is suggested that they should only be used as a secondary criterion.

In analogy to the publications by Hoffmann [8] and Köstermeyer [13], only two main technique groups should be distinguished here: a frontal one and a rotated one. However, the criteria for classification differ fundamentally. Hoffmann classifies a position as frontal when the face and the front of the body are oriented towards the wall. Köstermeyer considers a parallel shoulder axis to be a frontal characteristic. However, both the shoulder axis and the head have high degrees of rotational freedom as partial movements relative to the rest of the body. This complicates classification.

Therefore, the orientation of the pelvis relative to the climbing wall seems to be more suitable as a criterion. A crucial factor in this context is also the proximity of the pelvis to the CoM, which has a decisive influence on positioning while climbing. This additionally makes kinematic analyses easier to implement [59,60]. The greater trochanters and the superior iliac spines could serve as reference points for markers [61]; accelerometers can bring further usable data [62]. As a grouping criterion, the spatial relationship between the frontal plane of the pelvis and the plane of the climbing wall is proposed.

**Figure 3:**
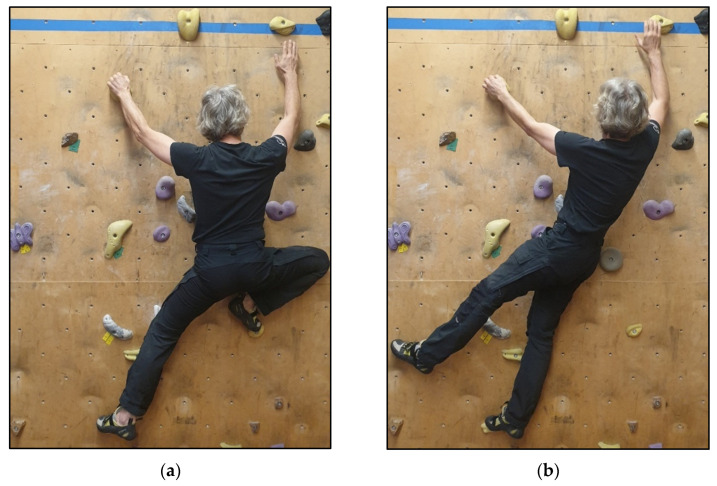
(**a**) frontal and (**b**) rotated climbing Λ-positions during phase 2 of a climbing movement.

In frontal climbing techniques, the frontal plane of the pelvis runs (almost) parallel to the climbing wall throughout all three phases of the technique. As a rule, when used functionally, the toes point in opposite directions (Figure 3a). This is considered a secondary criterion. In rotated climbing techniques, an angle of up to 90° is formed between the plane of the climbing wall and the pelvic plane, and the two planes intersect. This spatial relationship occurs in at least one phase of the climbing technique. The pelvic rotation takes place around the longitudinal axis (pirouette). As a secondary criterion, the toes often point in a similar direction (Figure 3b).

**Figure 4:**
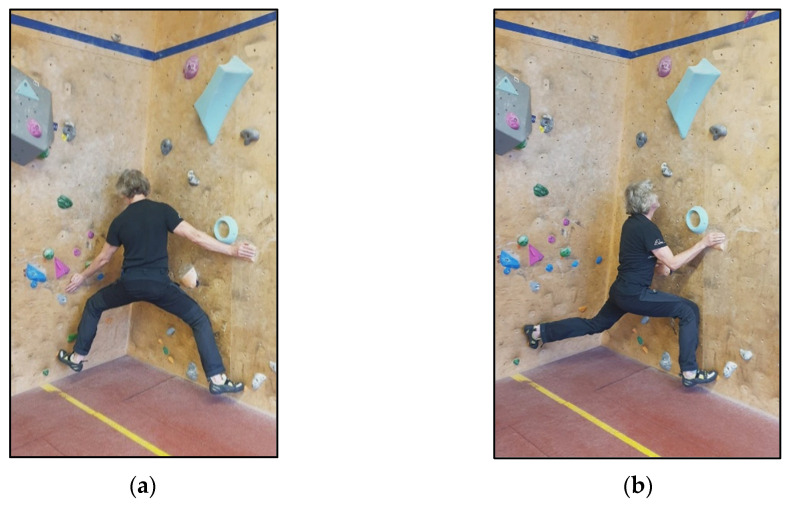
(**a**) frontal and (**b**) rotated climbing X-positions in a corner. Note: in (**a**) pelvic frontal plane intersects both wall surfaces; in (**b**), it is nearly parallel to the right one.

This classification cannot be directly transferred to climbing movements that take place on multiple walls (e.g., corners, chimneys, body cracks). The definition based on the spatial relationship of the planes is modified as follows: If the frontal plane of the pelvis intersects both climbing wall planes, the technique is classified as frontal (Figure 4a). If the climbing technique is performed such that the frontal plane of the pelvis runs (almost) parallel to one of the two climbing wall planes during a phase, it is categorized as rotated (Figure 4b).

#### 3.2.2. Examples of Climbing Techniques

In Table A1 in Appendix A, examples of frontal and rotated techniques are given with a systematic nomenclature. In the climbing jargon, the single techniques are termed differently. Some techniques can be performed frontally or rotated, e.g., hand cross. Others do not have an equivalent in the other category, like frogging. Sub-techniques and combinations are neglected for simplification. Two specific techniques per category are presented in terms of their partial movements (key characteristics) and linked to the climbing principles. The natural frontal gripping and stepping techniques have already been described extensively using the three-phase model (Figure 2). The economical X-position is characterized by a lowered CoM close to the wall, bent knees, and almost fully extended arms. The footholds are loaded via the big toes, which point outwards. Ideally, the handholds are gripped naturally (general grip) [7]. In the main phase (2) of the movement, one extremity is moved via the Υ- or Λ-positions into a new X-position (phase 3) [5,7]. Throughout the entire movement, the pelvic plane remains parallel to the climbing wall. This technique and the following ones can be viewed in explanatory videos provided in Supplementary Material (SM).

If different hold–foothold configurations are given, individual key characteristics of the movement change accordingly. If, during a movement to the upper right, the left handhold can only be loaded from the right side as a side pull, the principle of optimal wall contact again applies. In this case, it is no longer possible to pull on the hold with the right side; instead, the climber pushes away from the hold with the left one. The muscle slings required for this move (Λ-position) are now completely different from those with a natural grip, having high demands of the shoulder girdle muscles. This frontal technique is, therefore, referred to as “shoulder move” but also “Gaston move” [6].

The movements differ more significantly in the rotated techniques. The natural rotated gripping technique starts from a frontal X-position, the foot is placed on a foothold in or slightly outside of the vertical line of the handhold (side pull). This creates an unstable position (problem of the “barn door”). The free leg (stabilizing leg) counteracts the tendency to turn off the wall. At the same time, it helps to rotate the hip of the side of the mainly loaded foot towards the wall (CoM-shift). In this way Λ-position is efficient by applying the sub-principles of diagonality, three points of contact, and CoM-shift close to the wall. Subsequently, full stabilization is usually regained in a frontal X-position.

Another rotated technique, often referred to as Egyptian [7], also has the characteristics of a rotated pelvic axis and toes pointing in similar directions. During this technique, the main grip is ideally positioned as a side grip between both footholds. A rotated Λ-position is built up from a frontal X-position by rotating the pelvis towards the wall (CoM-shift) and pressing the two footholds apart from each other to increase tension. If one knee drops below the ipsilateral foot level and the patella points downwards, it is called “drop knee”. The position achieves stability during the Λ-position. Then, a frontal X-position is assumed again.

### 3.3. Hand and Foot Positioning

The alignment of the grip and foot surfaces influences the technique, exemplified by the shoulder move technique mentioned above. For a complete overview of alignments, it is therefore necessary to take a quick look at the holds and, secondarily, the way of loading by analyzing common finger and foot positions. The shape of the holds determines the type of grip (e.g., jugs, ledges, pinches, slopers). The orientation of the grip surface determines where the hold can be gripped. A distinction is made between top hand, undercling, side pull, Gaston (side pull for the other hand) and mixed forms. The shape and size of the grip surface determine how the hold can be gripped to establish an optimal force or form-fit. For that, finger positions are addressed next.

#### 3.3.1. Gripping Types—Finger Positioning

There is a broad consensus in the literature regarding which finger positions are suitable for which type of grip. However, the classification and terminology used are inconsistent [63,64,65,66,67,68]. Here, we propose classifying finger positions based on the finger joints involved = functional anatomy. This specifies which finger joints are flexed and which are extended. From distal to proximal, the distal interphalangeal joint (DIP), the proximal interphalangeal joint (PIP), and the metacarpophalangeal joint (MCP) are considered. Next, we look at which finger phalanges (Ph1 = proximal phalanges, Ph2 = medial phalanges, Ph3 = distal phalanges) are in contact with the grip surface and transfer the force. In this analysis, we do not consider clamping variations for cracks and special variations such as chisel, pinky mono, meat hook, or packing. Table A2 in the Appendix A lists open, half-open and closed finger-hand positions. The latter includes an example for a bent position and several crimp grips.

#### 3.3.2. Footwork—Foot Positioning

There is a broad consensus on how foothold surfaces should be loaded. The literature is very consistent in terms of terminology. A distinction is made between stepping with the toe (big toe), the inside and outside edge, as well as hooking [58]. Footholds are categorized as horizontal, sloped and incut ledges. Clamping footwork is omitted here, similar to finger positions. In addition to size, the orientation of the stepping surface is crucial. Footholds pointing upwards are typically loaded with the tiptoe, the inside, or the outside of the foot. Here, the technique category is decisive. For frontal techniques, stepping usually occurs with the toe pointing outward (see above). The load is on the big toe (distal phalanx). On large footholds, the proximal phalanx or even the base joint of the big toe can be in contact with the wall. For rotational techniques, one foot rotates accordingly, and wall contact occurs with the outside edge of the climbing shoe.

Two climbing principles are crucial for stepping on footholds: optimal CoM-shift and wall contact. Specific aspects should be addressed, such as sloped footholds. The optimizations of the two mentioned principles aim to maximize the static friction force (F_f_) to minimize the risk of slipping off.

F_f_ = μ · F_N_, (1)

where μ is the coefficient of static friction and F_N_ is the normal force, which acts perpendicular to the inclined surface. The coefficient is a constant and depends on factors such as material pairing (climbing shoe and wall), surface roughness, humidity and temperature. However, the static friction force can be directly influenced by maximizing the normal force. This is achieved by minimizing the distance between the contact point on the foothold and the action line of the weight force (lever arm), or simplified, the CoM needs to be above the foothold. This way, the weight force acts perpendicularly on the support surface, optimizing the normal force. The friction force increases proportionally. Simultaneously, this enhances the principle of optimal wall contact.

To additionally achieve an optimal form-fit connection, the normal force should be evenly distributed across the contact surface. This optimizes the area where friction can act and is comparable to wider tires on a car. The climbing shoe sole should be positioned parallel to the inclined foothold surface. The application of both main principles explains the climbing rule for sloped footholds: transfer CoM over the foothold and lower the heel. If the surface of the foothold is pointing sideways, force can also be applied by pulling on the foothold. This is typically done by using the heel (heel hooking) or the top of the toes (toe hooking). However, generating a pull through the shoe sole and “rocking over” the foothold might also be possible [6]. To get a video impression of the fundamental types of footwork and hooks, respectively, video links are provided in the SM.

## 4. Discussion and Conclusions

The aim of this work was to simplify the complexity of climbing movements to make them analyzable for sports scientists and practitioners. This framework focuses on the most crucial aspects but does not claim to be exhaustive. The systematization of holistic aspects of climbing skill is presented to the community for discussion and further development. Some practical recommendations in the context of training and performance diagnostics should be derived. A common language regarding climbing techniques and principles serves as the foundation for sports scientists, physical education teachers, coaches, and athletes to engage in professional exchange. This perspective paper provides suggestions for sports science structuring and definition.

Evaluation of the climbing skill for scientific purposes should be based on sport-specific physical fitness components (e.g., finger flexor strength) [16,17] and biomechanical principles (e.g., CoM-shift) [42,47,52]. The advantage lies in their measurability. For specific aspects like techniques and hand and foot positioning, expert ratings seem to be necessary. Data acquisition is highly subjective and uneconomical; however, AI might offer new options in the future.

For kinesiologic (physical education) purposes, instructors, coaches, or teachers should consider what aspects of their students’ climbing skill they wish to analyze and improve. By doing so, it might be easier to identify strengths and weaknesses. Consequently, they can apply the most suitable teaching methods. Additionally, transfer-effects and synergies can be promoted. These evaluations can target general components like physical fitness and climbing principles or specific techniques, footwork, and gripping types. For differentiation within this complex sport, body positioning can be separated from foot and hand positioning. Positioning, in turn, can be distinguished from movements. The three-phase model of climbing movements can be used to identify techniques and assign important characteristics to each phase [4,8,13]. Frontal techniques are recommended for beginners, while rotated ones are suited for advanced students. These can be simply distinguished by hip positioning: climbing with both hips facing the wall (frontal climbing technique) during all phases versus rotating one hip towards the wall during at least one phase (rotated climbing technique).

**Figure 5:**
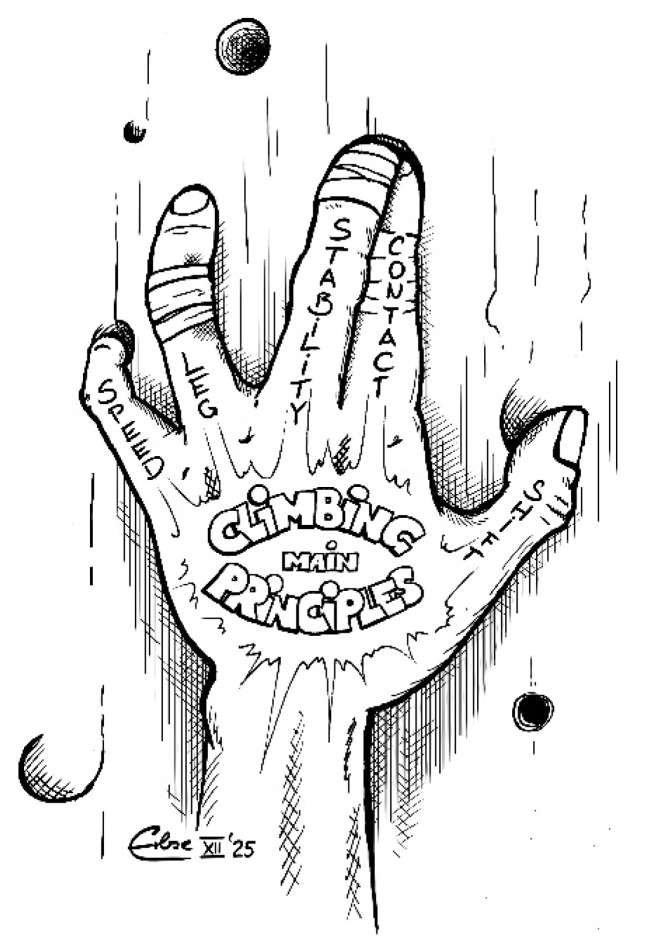
Overview of the five Main Climbing Principles in short form. With thankful permission from the illustrator.

Regarding the systematization of general aspects, five fundamental climbing principles have been identified. These are crucial for efficiency and safety in climbing. The principles—optimal wall contact, maintained stability, center of mass shift, movement initiation by the legs, and optimal climbing speed—offer climbers practical guidance for energy saving and performance. They are significant in preventing common climbing problems, such as the ‘Barn Door’ effect [6,8]. Figure 5 sketches the proposed five main climbing principles in short form: Stability, Contact, Shift, Leg, and Speed. This cartoon presents a method of recalling these fundamentals by looking at the tattooed back of the hand before each climbing technique, which might facilitate simplified communication among coaches, sports scientists, and athletes. Future studies could focus on how these principles are applied by beginners versus advanced climbers in order to develop targeted training approaches.

## Data Availability

No new data were created or analyzed in this study. Data sharing is not applicable to this article.

## Supplementary Materials

The following supporting information can be downloaded at: https://www.mdpi.com/article/10.3390/jfmk11010103/s1.

## Appendix A

**Table A1:** Exemplary overview of climbing techniques categorized into frontal and rotated ones.

Frontal Climbing Techniques	Rotated Climbing Techniques
Natural Frontal Gripping	Natural Rotated Gripping
Natural Frontal Stepping	Egyptian
Back-Flagging	Inside-Flagging
Frontal Hand Crossing	Rotated Hand Crossing
Frontal Foot Crossing	Rotated Foot Crossing
Frontal Foot Switching	Rotated Foot Switching
Frontal Stemming	Rotated Stemming
Frogging	Figure of Four
Shoulder-Pull	Figure of Nine
Mantling	Lay Backing

Sub-techniques and combinations are neglected.

**Table A2:** Structure of gripping types based on finger joint positions and contact of phalanges with grip surface.

Gripping Type:	Closed Hand							
	Soft Crimp		Hard Crimp				Sharp Crimp	Bent
finger position	middle ledge	middle ledge	small ledge	small ledge	narrow pinch with small ledge	sloper	micro ledge	jug
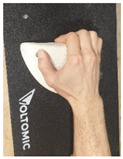
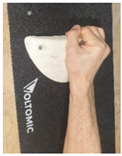
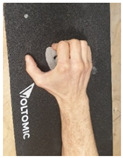
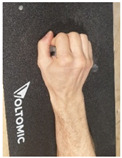
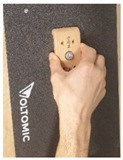
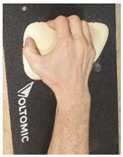
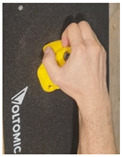
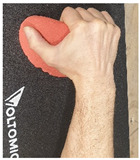
DIP	ex	ex	(over-)ex	(over-)ex	(over-)ex	(over-)ex	flex	flex
PIP	flex	flex	flex	flex	flex	flex	flex	flex
MCP	ex	ex	ex	ex	ex	ex	ex	flex
phalanges in wall contact	Ph3, Ph2	Ph3, Ph2 (thumb over DIP)	Ph3	Ph3 (thumb over Ph3)	Ph3 (opposing thumb)	Ph3 (adjacent thumb)	Ph3 (nail tip)	Ph3, 2, 1 (adjacent thumb)
**Gripping Type:**	**Open Hand**					**Half-Open Hand**		
finger position	small ledge	sloper	deep 2-finger pocket	flat 2-finger pocket	sloper wide pinch	edge	wide edge pinch	
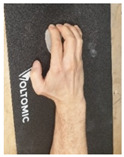
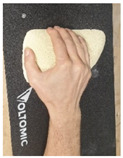
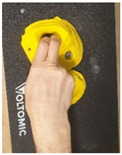
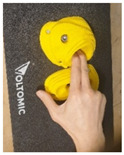
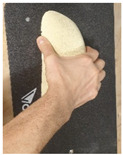
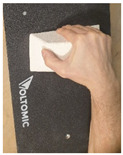
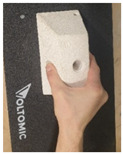
DIP	flex	flex	flex	flex	flex	ex	ex	
PIP	flex or ex (depending on finger length)	flex	flex	ex	flex	ex	ex	
MCP	ex	flex	ex	ex	flex	flex	flex	
phalanges in wall contact	Ph3	Ph3, 2, 1	Ph3, 2	Ph3	Ph3, 2, 1, thumb	Ph3, 2, 1	Ph3, 2, 1, thumb	

DIP—distal interphalangeal joint; PIP—proximal interphalangeal joint; MCP—metacarpophalangeal joint; flex—flexed; ex—extended; Ph1—proximal phalanx; Ph2—medial phalanx; Ph3—distal phalanx.
