# Supplementary material for: Biomechanical Principles and Techniques—A Systematization for Sport Climbing
Source: J Funct Morphol Kinesiol. 2026 Feb 28;11(1):103. doi: 10.3390/jfmk11010103 (PMC13027491; doi:10.3390/jfmk11010103)
Supplement: Supplementary file 1 [file jfmk-11-00103-s001.zip › jfmk-4117457-supplementary.pdf]

Journal of
Functional Morphology
and Kinesiology

MDPI

Supplementary Material

“Biomechanical Principles and Techniques – A Systematization for Sport Climbing.”

Silas Dech and René Kittel

Video examples of climbing techniques in the order as stated in the text.

Natural frontal gripping and stepping techniques:
https://www.youtube.com/watch?v=wSrk4Y4tf-A.

Shoulder move:
https://www.youtube.com/watch?v=jNsgZQsMgm0.

natural rotated gripping technique
https://www.youtube.com/watch?v=FG3Y9QcARB4.

Egyptian
https://www.youtube.com/watch?v=Yp2xr23NdGI

Further techniques can be found in explanatory videos on this channel https://www.youtube.com/@J_T_UP/videos.

Fundamental types of footwork and hooks, respectively, can be viewed here:
https://www.youtube.com/watch?v=wuzw3xMVYDQ
https://www.youtube.com/watch?v=0ce44Mj8CjM.
